# Magnesium Limitation Leads to Transcriptional Down-Tuning of Auxin Synthesis, Transport, and Signaling in the Tomato Root

**DOI:** 10.3389/fpls.2021.802399

**Published:** 2021-12-23

**Authors:** Muhammad Ishfaq, Yanting Zhong, Yongqi Wang, Xuexian Li

**Affiliations:** ^1^Key Laboratory of Plant-Soil Interactions, College of Resources and Environmental Sciences, Ministry of Education, National Academy of Agriculture Green Development, China Agricultural University, Beijing, China; ^2^Department of Vegetable Sciences, China Agricultural University, Beijing, China

**Keywords:** magnesium limitation, MRS2/MGT gene family, auxin, auxin signaling, PIN family, root system

## Abstract

Magnesium (Mg) deficiency is becoming a widespread limiting factor for crop production. How crops adapt to Mg limitation remains largely unclear at the molecular level. Using hydroponic-cultured tomato seedlings, we found that total Mg^2+^ content significantly decreased by ∼80% under Mg limitation while K^+^ and Ca^2+^ concentrations increased. Phylogenetic analysis suggested that Mg transporters (MRS2/MGTs) constitute a previously uncharacterized 3-clade tree *in planta* with two rounds of asymmetric duplications, providing evolutionary evidence for further molecular investigation. In adaptation to internal Mg deficiency, the expression of six representative *MGT*s (two in the shoot and four in the root) was up-regulated in Mg-deficient plants. Contradictory to the transcriptional elevation of most of *MGT*s, Mg limitation resulted in the ∼50% smaller root system. Auxin concentrations particularly decreased by ∼23% in the Mg-deficient root, despite the enhanced accumulation of gibberellin, cytokinin, and ABA. In accordance with such auxin reduction was overall transcriptional down-regulation of thirteen genes controlling auxin biosynthesis (*TAR*/*YUCs*), transport (*LAXs, PINs*), and signaling (*IAAs, ARFs*). Together, systemic down-tuning of gene expression in the auxin signaling pathway under Mg limitation preconditions a smaller tomato root system, expectedly stimulating *MGT* transcription for Mg uptake or translocation.

## Introduction

Magnesium (Mg), a non-substitutive component, performs a vast array of physiological and biochemical functions in plants. The well-addressed function of Mg is its participation in photosynthetic CO_2_ assimilation (Gerendás and Führs, 2013; Li J. et al., 2020). A large proportion (15–35%) of total available Mg within a plant is confined to the light-capturing complex of chloroplasts, where it not only functions as a structural element of chlorophyll (Chl) but also underpins photosynthetic performance (Farhat et al., 2016; Chen et al., 2018). Mg also acts as a cofactor of various enzymes (>300) involved in essential biological processes such as Chl biosynthesis, sugar transportation, and energy metabolism (Ma et al., 2016; Chen et al., 2018; Koch et al., 2019). Recent meta-analysis-based studies highlighted that Mg deficiency is becoming a rising concern in most production systems due to the large removal of Mg in intensive crop production systems and injudicious fertilization (Hauer-Jákli and Tränkner, 2019; Wang et al., 2020).

Plant roots employ adaptive mechanisms in response to environmental stimuli or nutrient variations. For instance, mild nitrogen or phosphate deficiency tends to enhance root growth for a greater nutrient absorption or translocation system (Ahmad et al., 2018; Meier et al., 2020; Nadeem et al., 2020). However, how roots respond to the Mg deficiency stress is inconsistent: root growth is clearly reduced by Mg deficiency in bean plants (*Vicia faba* L.) (Neuhaus et al., 2014), *Arabidopsis thaliana* (Gruber et al., 2013; Li D. et al., 2020), and potato (*Solanum tuberosum* L.) (Koch et al., 2019, 2020). However, in other studies, Mg deficiency does not considerably affect root growth in *Arabidopsis* (Hermans and Verbruggen, 2005) and Chinese cabbage (Verbruggen and Hermans, 2013). Notably, low Mg supply leads to more root hair development in *Arabidopsis* as an adapted strategy (Niu et al., 2014; Liu M. et al., 2018). Hence, further study is required to unravel root adaptation to Mg deficiency stress at morphological and molecular levels in different plant species, especially in model crop plants.

Mg transporter genes, homologues of CorA Mg transporters, have been identified across crop plants and classified into the CorA/MRS2/MGT family (Chen et al., 2018; Yan et al., 2018; Zhang et al., 2019a). MRS2/MGT genes are supposed to be primary transporters for Mg^2+^ uptake, distribution, and homeostasis in plants (Li et al., 2001, 2016; Marschner, 2012; Saito et al., 2013). In *Arabidopsis*, the MRS2/MGT gene family is composed of ten members, and several genes are important for Mg^2+^ uptake and transportation under normal or Mg limitation (Oda et al., 2016; Yan et al., 2018). However, the activity of these *MGTs* relies on the genetic makeup of plant species (Cui et al., 2016). Mg deficiency alters MRS2/MGT expression in the rice root (Chen et al., 2017; Zhang et al., 2019b); however, moderate Mg deficiency had no impact on MRS2/MGT transcription in the *Arabidopsis* root (Hermans et al., 2010; Ogura et al., 2018). Therefore, further molecular investigation is needed to understand the evolutionary dynamics, and transcription patterns of these MRS2/MGT family transporters in plant roots under Mg limitation.

Auxin is a key player regulating root growth and development (Porco et al., 2016; Stoeckle et al., 2018; Meier et al., 2020; Moret et al., 2020; Hu et al., 2021). During auxin biosynthesis, TRYPTOPHAN AMINOTRANSFERASE OF ARABIDOPSIS/TRYPTOPHAN AMINOTRANSFERASE-RELATED (TAA/TAR) controls indole-3-pyruvic acid (IPA) generation and YUCCA (YUC) acts to convert IPA into Indole-3-acetic acid (IAA) (Mashiguchi et al., 2011; Song et al., 2017). Auxin then moves across membranes by AUXIN1/LIKE-AUX1 (AUX1/LAX) influx carriers, PIN-FORMED (PIN) efflux carrier, PIN-LIKES (PILS), or ATP-binding cassette subfamily B (ABCB)-type transporters (Sauer and Kleine-Vehn, 2019; Vosolsobe et al., 2020). PIN transporters largely control intercellular and intracellular auxin transport (Zhang Y. et al., 2020); LAX3 acts as an auxin influx carrier during root development (Maghiaoui et al., 2020). Finally, AUXIN RESPONSE FACTORS (ARFs) together with AUXIN/INDOLE ACETIC ACID (Aux/IAA) regulate downstream gene expression in the auxin signaling pathway (Roosjen et al., 2018). Under low availability of IAA, Aux/IAA proteins bind to AUXIN RESPONSE FACTORS (ARFs) to prevent their expression (Lavenus et al., 2013; Mironova et al., 2017); while higher levels of IAA promotes Aux/IAA degradation to release the suppression of auxin-responsive genes (Wang and Estelle, 2014; Weijers and Wagner, 2016). Such Aux/IAA degradation is strongly linked with lateral root development (Guseman et al., 2015).

Auxin biosynthesis, transport, and signaling are essential components related to root growth and root system architecture. Nutrient deficiency stresses frequently enhance auxin accumulation in roots. For instance, low levels of nitrate stimulate auxin accumulation in roots of *Arabidopsis* and maize (Ma et al., 2014; Sun et al., 2020), and low concentrations of ammonium also increase auxin content in the *Arabidopsis* root (Meier et al., 2020). Similarly, phosphorus deficiency improves auxin levels in roots of *Arabidopsis* and Foxtail millet (Ahmad et al., 2018; Bhosale et al., 2018), and potassium limitation modulates the expression of auxin-responsive genes in the root of rice (Ma et al., 2012). Zinc supply alters auxin homeostasis in the roots of *Arabidopsis* (Zhang et al., 2018; Wang et al., 2021). Few studies show the distribution and transcriptional variations of auxin in response to Mg supply. For instance, P and Mg interactively affect the redistribution and accumulation of auxin in *Arabidopsis* roots through altered signaling functions of *AUX1*, *PIN2*, and *PIN3*, which modulate primary root elongation and growth direction (Niu et al., 2015). In another study, optimum Mg supply promotes PIN2-based polar auxin transport and distribution under aluminum toxicity, which restores root growth by regulating root surface pH (Zhang Z. et al., 2020). Transcriptomic data show that Mg deficiency differentially regulates expression of auxin-responsive genes in *Arabidopsis* and *Citrus sinensis* (Hermans et al., 2010; Yang et al., 2019). For instance, Mg deficiency altered expression of auxin efflux carrier proteins and AUX/IAA (*IAA1*, *IAA5*, *IAA6*, *IAA14*, *IAA17*, *IAA19*) in the leaves of *Arabidopsis* (Hermans et al., 2010). Similarly, under Mg deficiency, *IAA11*, *IAA13*, *IAA29*, and *ARF4* are upregulated by 2-, 1. 5-, 2. 8-, and 2.5-fold, respectively, in the leaves of *Citrus sinensis* (Yang et al., 2019). However, how Mg limitation affects auxin homeostasis and related gene expression in plant roots remains elusive.

Tomato (*Solanum lycopersicum* L.) is one of the most important vegetable plants worldwide and it requires more Mg to form the same biomass than grass or grain crops (Broadley and White, 2010; Gerendás and Führs, 2013). In this study, we used tomato as a model plant to investigate the response of seedlings, especially the root system, to low external Mg supply at the molecular level. We performed the evolutionary analysis of the MRS2/MGT-type Mg transporter family for further molecular investigation. We found overall up-regulation of Mg transporters in the smaller root and consistent down-regulation of auxin accumulation and related gene expression in the root under Mg limitation.

## Materials and Methods

### Plant Growth Conditions and Experimental Set-Up

This study was conducted in a standard greenhouse at China Agricultural University, Beijing, China. The greenhouse environment was as follows: 28/22°C temperature, 60% relative humidity, 14/10 light/dark photoperiod, and natural daylight. Tomato (*Solanum lycopersicum* L.) cv. Xianliang, procured from Dalian Lida Seed Company (China), was used as the plant material in this study. Seeds were first surface-sterilized by heating in a water bath at 55°C for 15 min, and soaking in 10% Na_3_PO_2_ for 20 min. Seeds were then nurtured by imbibing in distilled water (DW) for 6 h, and germinated on moist filter paper covered with black plastic wrap in a growth chamber. The sprouted seeds with a 2-cm primary root were sown in 50-cell seedling plug trays filled with commercially available potting soil containing low indigenous Mg (46 mg kg^–1^), and were irrigated regularly with DW.

At the four fully unfolded compound leaf stage (∼23-days after sowing), consistent and uniformly sized seedlings were transferred into continuously aerated 10-L (in 28 × 25 × 17 cm pots) nutrient solution. Nutrient solution composition was slightly modified from a published protocol for tomato culture (Cámara-Zapata et al., 2019). The final nutrient solution contained macro- and micronutrients as follows: 4 mM Ca (NO_3_)_2_.4H_2_O, 0.6 mM KNO_3_, 1 mM KH_2_PO_4_, 1 mM MgSO_4_.7H_2_O, 90 μM Na_2_Fe-EDTA, 25 μM H_3_BO_3_, 2 μM MnSO_4_.H_2_O, 2 μM ZnSO_4_.7H_2_O, 0.5 μM CuSO_4_.5H_2_O, and 0.5 μM (NH_4_)_6_Mo_7_O_24_.4H_2_O. The experiment contained two levels of Mg, one control (Ctrl) having optimum Mg as 1 mM MgSO_4_.7H_2_O, and another low Mg (LMG) consisting of 0.02 mM MgSO_4_.7H_2_O. The LMG level was determined according to our preliminary phenotypic screen by growing tomato seedlings at varying Mg concentrations (0.02, 0.06, 0.25, 1, 4 mM). The onset of the treatment was established at the time of transplanting with the intention to avoid Mg accumulation in the vacuole at the very initial growth stages (Hauer-Jákli and Tränkner, 2019). In the base solution, MgSO_4_ was the only source of SO_4_^2–^, meanwhile, equivalent moles of Na_2_SO_4_ was applied to Mg-depleted plants to maintain the relatively constant cation/anion balance and compensate the SO_4_^2–^ deficiency.

To avoid osmotic shock, seedlings were first grown in the 50%-strength nutrient solution for 3 days, and then full-strength nutrient solution was supplied till harvest. This study was performed with six biological replicates and each biological replicate had six plants (technical replicates). The nutrient solution was replaced at an interval of 3 days to avoid ion depletion. The pH was maintained at 6.0 with 1M TRIS (C_4_H_11_NO_3_). After a 3-week treatment, seedlings were dissected at the root-shoot junction, flash-frozen in liquid-N_2_, and stored at –80°C for physiological and molecular analysis. For root growth analysis, 2-week samples were also harvested. The experiment was reproduced at least two times.

### Elemental Analysis and Root-to-Shoot Translocation of Mg^2+^

For elemental analysis (Mg^2+^, K^+^, Ca^2+^, and Na^+^) in the plant material, oven-dried root and shoot samples were grounded separately by an electric grinder. The 100 mg of tissue powder was weighed using a high-accuracy balance, and transferred in a Teflon digestion tube. Followed by, 6 ml concentrated HNO_3_ was added and stood overnight until the vigorous reaction phase was over. Then, 2 ml H_2_O_2_ was added and digested in the microwave digestion system (MARS 6, CEM Microwave Technology LTD, United States). The digested solution was transferred into a 25−ml volumetric flask and filled up to the calibration mark with ultrapure water. The concentrations of minerals in the solution were determined by an inductively coupled plasma-optical emission spectrometer (OPTIMA 3300 DV, Perkin-Elmer, MA, United States). After every 20 samples, the blank sample was included to ensure the accuracy of measurements. At least two sets of samples were run with six replicates, and concentrations of each element were quantified as described in our earlier study (Ishfaq et al., 2021). The total Mg^2+^ content and root-to-shoot translocation of Mg^2+^ were successively quantified as follow: Total Mg^2+^ content = [(root dry biomass × root Mg^2+^ concentration) + (shoot dry biomass × shoot Mg^2+^ concentration)], and root-to-shoot translocation of Mg^2+^ = [(shoot dry biomass × shoot Mg^2+^ concentration)/Total Mg^2+^ content] × 100%.

### Sequence Blast, Alignment, and Phylogenetic Construction

To retrieve the protein sequences in angiosperms, lycophytes, mosses, liverworts, algae and fungi, MRS2/MGT proteins in *Arabidopsis thaliana* were used as queries to blast in Phytozome^1^ and National Center for Biotechnology Information.^2^ The sequences were scored and aligned sequentially using the MUSCLE 3.6 program,^3^ GeneDoc 3.2^4^ (Nicholas, 1997), and CLUSTALX.^5^ The protein matrix was used to plot the MRS2/MGT family tree. The phylogenetic tree was mapped by the MEGA 6.0 program^6^ following the neighbor-joining method, and the bootstrap was analyzed in 1,000 replications. The gene structure was plotted by using GSDS.^7^ Gene accession numbers were listed in Supplementary Table 1.

### Plant Phenotyping and Root System Analysis

The root and shoot dry weight was determined after separately drying in the oven at 65°C till constant weight. The root-to-shoot ratio was calculated on a dry matter basis. Each sample had six independent biological replicates, and each replicate was averaged by two samples harvested from the same pot.

At harvest, the entire root system dissected from the root-shoot junction was rinsed three times by DW and stored at –4°C until scan analysis. Each root was placed on a transparent plastic slide and thoroughly combed apart in DW. The desktop scanner (Epson Perfection V850 Pro) was used to scan the roots at a resolution of 400 dpi. The scanned images were analyzed using the WinRHIZO software (version 5.0) (Regent Instruments Inc., Quebec City, QC, Canada) to quantify root indexes as described (Freschet et al., 2020). The relative root growth rate (RRGR) was computed as a given equation (Samal et al., 2010).


(1) $$RRGR=\left(\frac{\text{In}\left(TRL2/TRL1\right)}{t2-t1}\right)$$

Where, ln is natural log, *TRL* is total root length in cm and *t* is time of harvest in days; and the subscripts 1 and 2 refer to the 14 and 21 days of harvesting after transfer, respectively.

### Extraction and Quantification of Endogenous Hormones

To analyze dynamic hormonal distribution under Mg limitation, enzyme-linked immunosorbent assay (ELISA) was performed having 96-well microtitration plates filled with extract of plant tissues or hormone standards and corresponding antibodies, according to the standard protocol (EL310, Bio-TEK, Winooski, VT) and the published method (Dai et al., 2009). Briefly, ∼0.5 g frozen plant tissue was fine grounded in a pre-cooled mortar, and homogenized in 10 ml 80% (v/v) methanol extraction medium containing 1 mM butylated hydroxytoluene (BHT) as an antioxidant. Subsequently, the collected extract was incubated at 4°C for 4 h, and then centrifuged at 4,000 rpm for 20 min at 2–4°C. The supernatant was passed through Chromosep C18 columns (C18 SepPak Cartridges, Waters, Millford, MA), prewashed with absolute methanol. The hormonal fraction was eluted with 10 ml of 100% (v/v) methanol and then 10 ml ether. The elute was then N_2_-dried, and dissolved in 2.0 ml phosphate-buffered saline (PBS) containing 0.1% (w/v) gelatin and 0.1% (v/v) Tween-20 (pH 7.5). The concentrations of IAA (indole-3-acetic acid), ABA (abscisic acid), GA3 (gibberellic acid 3), and ZR (zeatin riboside) were quantified at an optical density of *A*490 (Weiler et al., 1981).

### RNA Extraction and Quantification by RT-qPCR

Total RNA was extracted from ∼100 mg powdered (in liquid N_2_) tomato samples using the Trizol reagent, following the manufacturer’s instructions (Invitrogen). The quality of extracted RNA was checked by a NanoDrop Spectrophotometer. Next, 4–5 g RNA was digested by DNase 1 (Takara Biomedicals, Kyoto, Japan) to eliminate potential DNA contamination. Reverse transcription of RNA samples into cDNA was carried out using M-MLV reverse transcriptase (Thermo Fisher Scientific, Waltham, MA United States). Quantitative PCR (RT-qPCR) in a Bio-Rad iCycler iQ5 system (Bio-Rad, Hercules, CA, United States) was operated to quantify relative gene expression by using cDNA, SYBR Premix Ex Taq™ (Takara), and designed primers of genes of interest (Supplementary Table 2). The qPCR was programmed for 10 min of pre-incubation at 95°C, 40 cycles of denaturation at 95°C for 15 s, annealing at 60°C for 30 s, and extension at 72°C for 30 s. Four biological and two technical replicates were analyzed for each gene. The housekeeping gene *Slubi3* was used as an internal control (Liu S. et al., 2018). The equation 2^-(ΔΔCt) was used to determine relative gene expression (Livak and Schmittgen, 2001).

### Statistical Analysis

Data processing with simple calculations (e.g., mean, standard deviation [SD], maximum and minimum) was performed by Microsoft Excel-2019. One-way analysis of variance (ANOVA) along with the Tukey honest significant difference (HSD) test was performed by Statistix 8.1 (Analytical Software, Tallahassee, FL, United States) to analyze the statistical difference (*P < 0.05, **P < 0.01, ***P < 0.001) across treatments, as specified in figure legends. Bar graphs were plotted by GraphPad Prism 9.

## Results

### Elemental Homeostasis Under Magnesium Limitation

**FIGURE 1:**
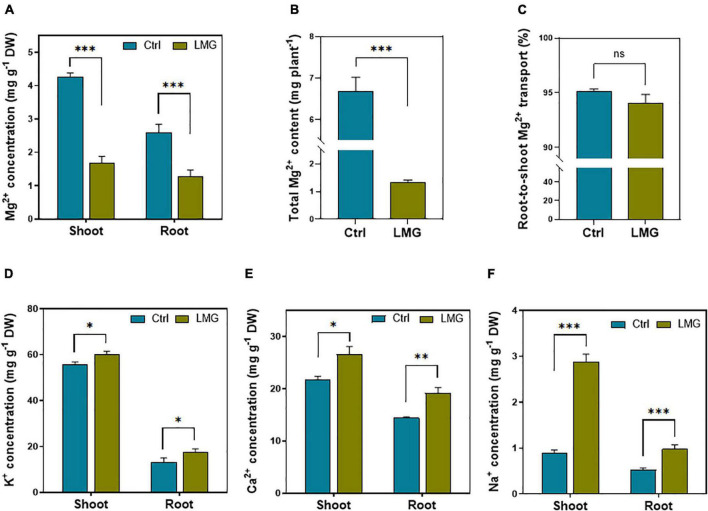
Mg limitation altered Mg^2+^ uptake and elemental homeostasis in tomato seedlings. **(A)** Mg^2+^ concentration in shoot and root (mg g^–1^ DM), **(B)** total Mg^2+^ content (mg pot^–1^), **(C)** root-to-shoot translocation of Mg^2+^ (%), **(D)** K^+^ concentration in shoot and root (mg g^–1^ DM), **(E)** Ca^2+^ concentration in shoot and root (mg g^–1^ DM), and **(F)** Na^+^ concentration in shoot and root (mg g^–1^ DM). The bar graph showed the mean value while whiskers represented the maximum/minimum values of six independent biological replicates. Asterisks indicated significant differences at *P < 0.05, **P < 0.01, and ***P < 0.001, according to Tukey’s HSD test. Where, Ctrl, control; LMG, low Mg; ns, non-significant.

The data of elemental homeostasis showed an obvious variation among treatments. The Mg^2+^ concentration significantly reduced from 2.6 to 1.29 mg g^–1^ (∼50%) in the tomato root, and 4.27–mg g^–1^ (∼61%) in the shoot under Mg limitation, compared to control (Figure 1A). The total Mg^2+^ content in the Mg-sufficient seedling was significantly higher by ∼80%, compared to LMG plants (Figure 1B). Out of whole plant Mg^2+^ uptake, we did not find significant variations in percent root-to-shoot translocation of Mg^2+^ among treatments (Figure 1C). Importantly, during Mg limitation, the concentration of other competing cations such as K^+^, Ca^2+^, and Na^+^ significantly increased by 34, 33, and 85% in the root, respectively. In the shoot, K^+^, Ca^2+^, and Na^+^ concentrations significantly increased by 9, 22%, and ∼2-fold, respectively, compared to control (Figures 1D–F).

### Evolutionary Analysis of the MRS2/MGT-Type Magnesium Transporter Family and MGTs Expression in Response to Magnesium Limitation

**FIGURE 2:**
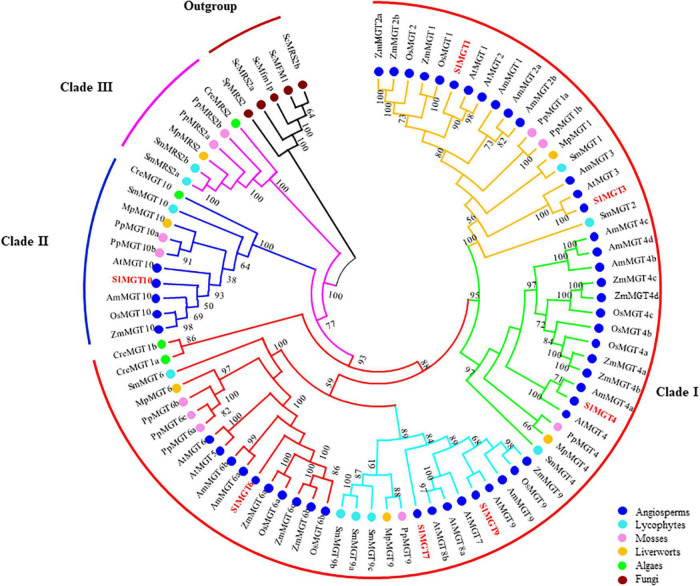
Phylogenetic analysis of the MRS2/MGT gene family in angiosperms, lycophytes, mosses, liverworts, algae and fungi. Three basal clades were indicated by red (Clade 1), blue (Clade II), and purple (Clade III) curves. In Clade 1, MGT1/2/3, MGT4/5, MGT7/8/9, and MGT5/6 branches were in yellow, green, light blue, and red, respectively. Numbers on branches indicated bootstrap values above 50.

**FIGURE 3:**
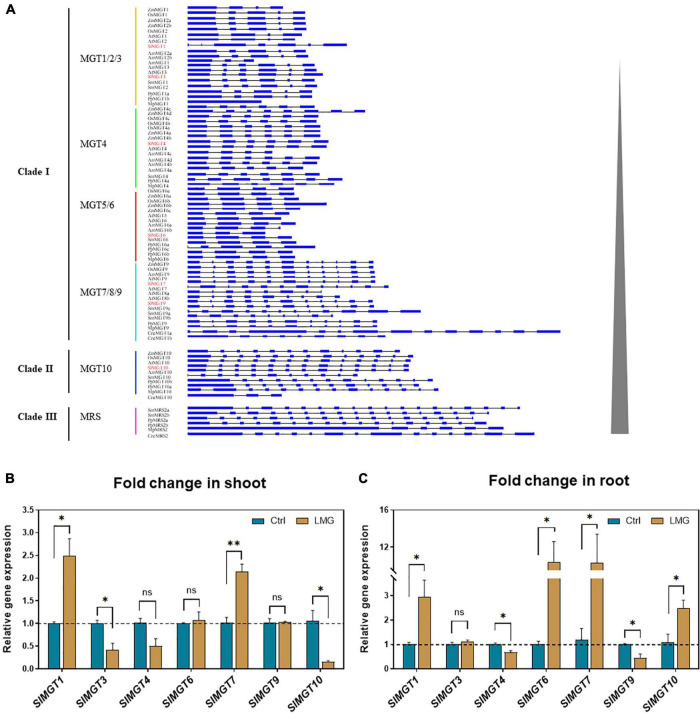
The gene structure of *MGTs*, and transcription patterns of *MGTs* in LMG seedlings. **(A)** Gene structure of MRS2/MGT-type Mg transporters, **(B)** relative gene expression of *MGTs* in the shoot, and **(C)** relative gene expression of *MGTs* in the root. The values of *MGTs* were reported as relative fold change from control, which was normalized to 1 as shown by dashed horizontal lines. The bar graph showed the mean value while whiskers represented the maximum/minimum values of four independent biological replicates. Asterisks indicated significant differences at *P < 0.05 and **P < 0.01, according to Tukey’s HSD test. Where, Ctrl, control; LMG, low Mg; *MGT*, Mg transporter; ns, non-significant.

To better select and analyze expression levels of representative Mg transporters, we constructed the phylogenetic tree of the MRS2/MGT-type Mg transporter family by retrieving corresponding protein sequences of *Solanum lycopersicum, Arabidopsis thaliana*, *Oryza sativa*, *Zea mays*, *Amborella trichopoda*, *Selaginella moellendorffii*, *Physcomitrella patens*, *Marchantia polymorpha*, and *Chlamydomonas reinhardtii*, with fungal MRSs as an out-group. Phylogenetic analysis showed that MRS2/MGT constitutes a previously uncharacterized 3-basal clade *in planta* with two rounds of asymmetric duplications. Briefly, Clade I split into four subclades (MGT1/2/3, MGT4, MGT5/6, MGT7/8/9), and Clade II(MGT1/2/3, MGT4the MGT10 subfamily. Clade I and Clade II contained members across angiosperms, lycophytes, mosses, liverworts, and algae, while Clade III had no members from angiosperms (Figure 2). The gene structure of the MRS2/MGT family also supported such a three-clade pattern, as shown in Figure 3A. The average exon number showed obvious changes across clades I, II, and III and average exon 1–12, 11–14, 6–18 exons, respectively. Less exons of MGT1/2/3, MGT4, and MGT5/6 are probably due to intron losses in these subfamilies during evolution.

**FIGURE 4:**
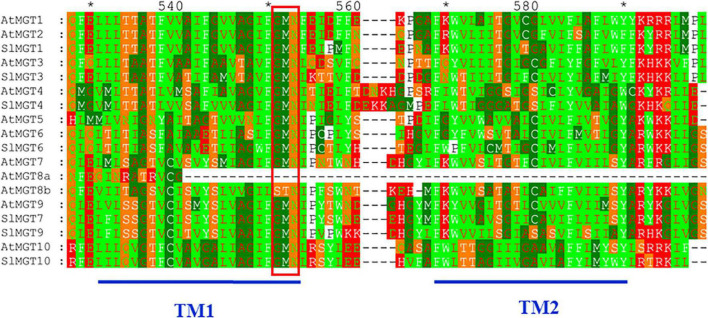
The alignment of *SIMGT* and *ATMGT* proteins. Conserved residues of each group of MRS2/MGTs were shaded in different colors. GMN (Gly-Met-Asn) tripeptide motif was indicated by red bar. Dashes represented gaps in the sequence and conserved residues that constituted the active sites were marked by asterisk. Numbers above the alignment gave the position of an amino acid within the alignment. Where, TM, transmembrane localization.

We suspected that reduced Mg accumulation in LMG seedlings was primarily attributed to differential expression (and further functioning) of Mg transporters. RT-qPCR results showed that Mg limitation stimulated expression *MGT1* and *MGT7* by 2.5- and 2.1-fold, respectively, in the shoot. Meanwhile, *MGT3* and *MGT10* expression was significantly down-regulated to 0.42- and 0.16-fold, respectively, in the shoot of LMG seedlings (Figure 3B). In the root, *MGT1*, *MGT6*, *MGT7*, and *MGT10* had 2. 9-, 10. 4-, 10. 3-, and 2.5-fold higher levels of transcription under Mg limitation, respectively. Meanwhile, the expression of *MGT4* and *MGT9* was down-regulated to 0.68- and 0.44-fold, respectively, in the LMG root, as shown in Figure 3C. Further, the alignment of *SIMGT* and *ATMGT* proteins showed that all differentially expressed MRS2/MGTs in tomato had two typical transmembrane domains and a GMN (Gly-Met-Asn) tripeptide motif (Figure 4).

### Plant Biomass and Root Architectural Indexes of Low Magnesium Seedlings

**FIGURE 5:**
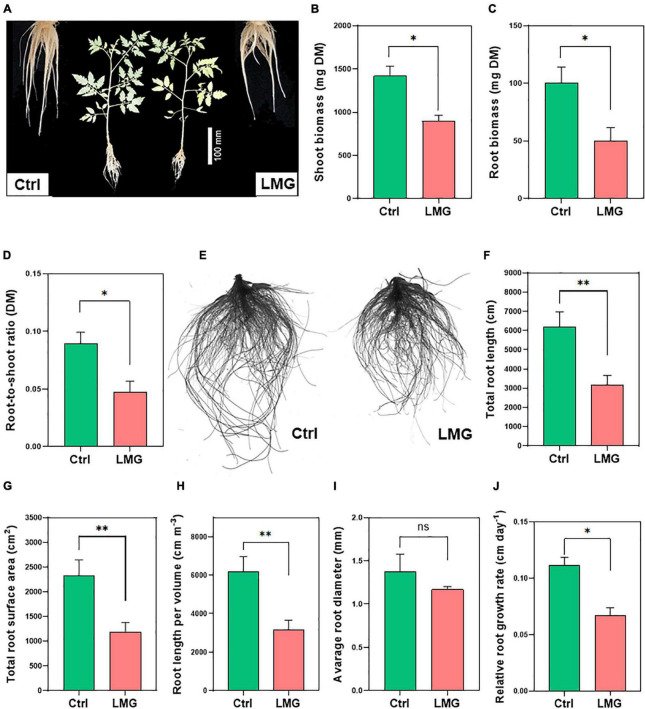
Mg limitation inhibited tomato growth and root architectural indexes. **(A)** Plant phenotypic responses after 3 weeks of treatments, **(B)** Root biomass (mg plant^–1^ DM), **(C)** Shoot biomass (mg plant^–1^ DM), **(D)** Root-to-shoot ratio (DM), **(E)** Scanned image of control and LMG treated roots **(F)** Total root length (cm), **(G)** Total root surface area (cm^2^), **(H)** Root length per volume (cm m^–3^), **(I)** Average root diameter (mm), and **(J)** Relative root growth rate (cm day^–1^). The bar graph showed the mean value while whiskers represented the maximum/minimum values of six independent biological replicates. Asterisks indicated a significant difference at *P < 0.05 and **P < 0.01, according to Tukey’s HSD test. Where, Ctrl, control; LMG, low Mg; ns, non-significant.

Mg limitation significantly reduced root and shoot dry biomass compared to control, with ∼50 and 36% decreases, respectively (Figures 5A–C). Interestingly, the percentage reduction in root biomass was higher compared to that in shoot biomass, which contributed to a ∼47% decrease of the root/shoot ratio of Mg-depleted plants, compared to control plants (Figure 5D).

Entire roots were scanned to examine treatment effects on root system architecture (Figure 5E). Root morphological indexes revealed a significant (*P* < 0.01) reduction in total root length (∼49%), root surface area (∼50%), and length per volume (∼49%) in Mg-depleted plants, compared to control (Figures 5F–H). Whereas, average root diameter (mm) did not show significant differences as shown in Figure 5I. We also observed a sharp decline in the overall root system after the 14-day treatment (Supplementary Figure 1). To better understand the dynamic change of root growth, we computed relative root growth rate (RRGR) by harvesting tomato seedlings on day 14 and 21 after treatments. Results showed 0.07 cm day^–1^ root growth under Mg limitation in contrast to 0.11 cm day^–1^ growth of control roots (Figure 5J).

### Magnesium Deficiency-Induced Hormonal Distribution and Expression Patterns of Auxin Biosynthesis

**FIGURE 6:**
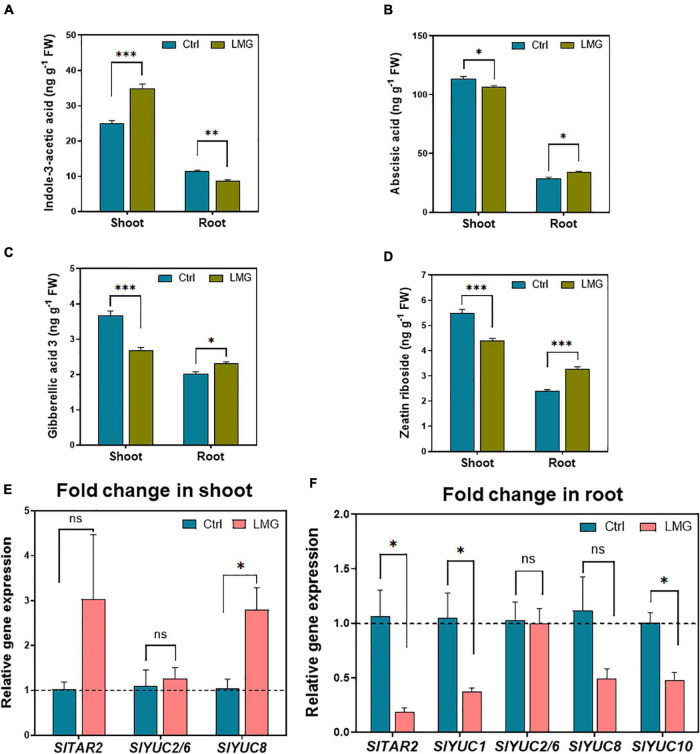
LMG disturbed the hormonal distribution and expression patterns of auxin biosynthesis in the root. **(A)** Indole-3-acetic acid concentration in shoot and root (ng g^–1^ FW), **(B)** Abscisic acid concentration in shoot and root (ng g^–1^ FW), **(C)** Gibberellic acid 3 concentration in shoot and root (ng g^–1^ FW), **(D)** Zeatin riboside concentration in shoot and root (ng g^–1^ FW), **(E)** Relative gene expression of *TAR*/*YUCs* in the shoot, **(F)** Relative gene expression of *TAR*/*YUCs* in the root. The bar graph showed the mean value while whiskers represented the maximum/minimum values of four independent biological replicates. Asterisks indicated significant differences at *P < 0.05, **P < 0.01, and ***P < 0.001, according to Tukey’s HSD test. Where, Ctrl, control; LMG, low Mg; ns, non-significant; YUC, YUCCA.

Below- and above-ground hormonal accumulation differed apparently under LMG, as shown in Figures 6A–D. The concentration of endogenous indole-3-acetic acid (IAA) was 40% higher (increased from 25 to 35 ng g^–1^) in the shoot of Mg-deficient plants. However, the IAA concentration surprisingly decreased by 23% (to 8.69 ng g^–1^) in the root of Mg-deficient plants, compared to control plants (Figure 6A), possibly due to diminished shoot-to-root IAA translocation under Mg limitation. Contradictory to the IAA accumulation pattern, other hormones displayed significantly higher accumulation in the root as compared to that in the shoot under Mg limitation: concentrations of ABA, GA3 and ZR reduced by 18, 27, and 20%, respectively, in LMG shoot. Meanwhile, approximately 6, 14, and 36% more ABA, GA3, and ZR, respectively, were detected in the LMG root, compared to control (Figures 6B–D).

In LMG shoot, the *YUC8* was upregulated by 2.8-fold and the expression of other auxin synthesis related genes, i.e., *TAR2*, *YUC2/6*, and *YUC8* remained unchanged (Figure 6E). Consistent with lower IAA concentrations in LMG roots, *TAR2*, *YUC1*, *YUC8*, and *YUC10* showed 0. 18-, 0. 37-, 0. 49-, and 0.48-fold down-regulation in the root under Mg limitation. However, *YUC2/6* and *YUC8* showed no significant change in expression (Figure 6F).

### Transcriptional Alterations of Auxin Transport and Signaling Genes Under Magnesium Limitation

**FIGURE 7:**
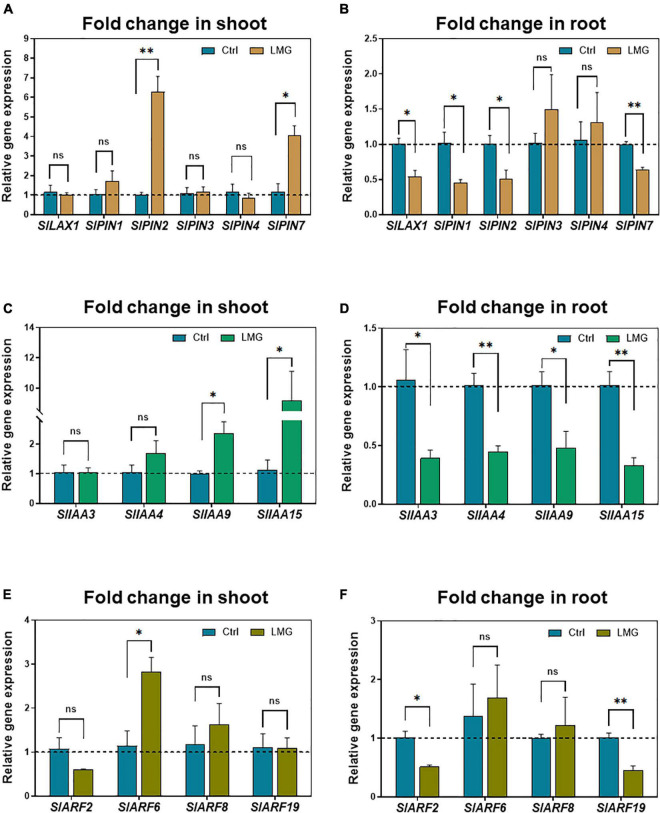
Transcriptional alterations of auxin transport and signaling genes under Mg limitation. **(A)** Relative gene expression of *LAX/PINs* in the shoot, **(B)** Relative gene expression of *LAX/PINs* in the root, **(C)** Relative gene expression of *IAAs* in the shoot, **(D)** Relative gene expression of *IAAs* in the root, **(E)** Relative gene expression of *ARFs* in the shoot, **(F)** Relative gene expression of *ARFs* in the root. The values were reported as relative fold change from control, which was normalized to 1 as shown by dashed horizontal lines. The bar graph showed the mean value while whiskers represented the maximum/minimum values of four independent biological replicates. Asterisks indicated a significant difference at *P < 0.05, **P < 0.01, according to Tukey’s HSD test. Where, Ctrl, control; LMG, low Mg; ns, non-significant; LAX, LIKE-AUX1; PIN, PIN-FORMED; Aux/IAA, AUXIN/INDOLE ACETIC ACID; ARFs, AUXIN RESPONSE FACTORS.

To further investigate the potential implications of auxin signaling in modulating root growth under Mg limitation, we quantified the expression of genes regulating auxin transport and signaling by RT-qPCR. With regard to auxin transport, *PIN2* and *PIN7* showed higher expression levels in the LMG shoot compared with control. No significant variation in expression of *LAX1*, *PIN1*, *PIN3*, and *PIN4* was observed in the Mg-depleted shoot (Figure 7A). In the LMG root, transcription of *LAX1*, *PIN1*, *PIN2*, and *PIN7* was significantly repressed to 0. 54-, 0. 45-, 0. 51-, and 0.64-fold, respectively, compared with control. However, *PIN3* and *PIN4* showed no significant change in expression (Figure 7B).

Beyond auxin transport is its signaling that orchestrates complicated physiological outcomes (Lv et al., 2020; Meier et al., 2020; Hu et al., 2021), therefore we analyzed expression of related *IAA* and *ARF* genes. Mg limitation led to significant up-regulation of *IAA9* and *IAA15* by 2.35- and 9.2-fold, respectively, in the LMG shoot (Figure 7C) in contrast to down-regulation of *IAA3*, *IAA4*, *IAA9*, and *IAA15* by 0. 39-, 0. 45-, 0. 48-, and 0.33-fold, respectively, in the LMG root (Figure 7D). Lastly, ARF genes had differential expression in the shoot and root under Mg limitation, too. *ARF6* had 2.8-fold higher expression in the LMG shoot, with no change in expression of *ARF2*, *ARF8*, and *ARF19*, compared with control (Figure 7E). Different from expression patterns in the shoot, no ARF expression was stimulated by Mg limitation in the root, while *ARF2* and *ARF19* expression was down-regulated by 0.51- and 0.45-fold, respectively, compared to control (Figure 7F).

## Discussion

Mg plays fundamental roles in regulating crop production and produce quality, and Mg deficiency is emerging as an increasing agricultural and nutritional issue required to be tackled worldwide (Hauer-Jákli and Tränkner, 2019; Wang et al., 2020; Fiorentini et al., 2021). However, how plants adapt to Mg deficiency stress remains largely unclear or inconsistent. Here, we presented a previously uncharacterized 3-clade tree for Mg transporters *in planta*, revealed up-regulation of six representative *MGT*s in Mg-deficient tomato seedlings, and characterized a smaller root system harboring coherent down-regulation of auxin accumulation and expression of related genes in auxin signaling in an important horticultural model crop, providing new valuable insights for future studies.

### Differential Expression of MGTs in Tomato Seedlings Favors Mg^2+^ Uptake and Translocation in Response to Low Magnesium

In our study, tomato seedlings suffered from Mg deficiency from the first day of transplanting into the nutrient solution which may avoid Mg^2+^ accumulation in the vacuole during initial growth stages (Hauer-Jákli and Tränkner, 2019). Mg limitation reduced Mg^2+^ uptake by tomato roots (Figures 1A,B). Low eternal Mg conditions also disturbed elemental homeostasis by increasing uptake of other competing cations, i.e., K^+^, Ca^2+^, and Na^+^. To analyze expression of representative *MGTs*, we generated a unified three-clade phylogenetic tree of the MRS2/MGT gene family (Figure 2), which provided the evolutionary foundation for future functional characterization of *MGTs*. In *Arabidopsis*, MGT6 regulates cellular homeostasis of Mg^2+^ under Mg limitation (Mao et al., 2014; Oda et al., 2016; Yan et al., 2018). Mg limitation results in higher *MGT1* expression in *Arabidopsis* (Lenz et al., 2013) and rice (Zhang et al., 2019b). In tomato, all differentially expressed *MGTs* fell into clade I except MGT10 in clade II (Figures 2, 3A). In total, six *MGTs* showed differential expression between control and LMG seedlings, and four up-regulated (*MGT1*, *MGT6*, *MGT7*, and *MGT10*) transporters in root probably favored Mg^2+^ uptake and translocation (Figure 3C). Differential expression of *MGT1*, *MGT3*, *MGT7*, and *MGT10* in the shoot possibly manipulate Mg^2+^ translocation within plant tissues under Mg limitation (Figure 3B). The relative functional importance of these *MGTs* in tomato adaptation to Mg limitation calls for further studies to unravel underlying molecular mechanisms.

### Transcriptional Down-Tuning of Genes Regulating Auxin Biosynthesis, Transport, and Signaling Conditioned a Smaller Seedling Root Under Magnesium Limitation

Root growth is largely programmed by internal genetic, developmental cues and various environmental stimuli primarily via hierarchical hormone signaling cascades (Lakehal and Bellini, 2019; Alaguero-Cordovilla et al., 2021; Hu et al., 2021). Auxin plays a central role in root initiation, elongation, and architectural configuration (Olatunji et al., 2017; Meier et al., 2020; Sun et al., 2020). *YUCs* overexpression promotes IAA production in *Arabidopsis* (Mashiguchi et al., 2011; Novak et al., 2012). Here, *TAR2*, *YUC1*, *YUC8*, and *YUC10* downregulated by 0. 18-, 0. 37-, 0. 49-, and 0.48-fold in Mg-depleted roots (Figure 6F), suggesting that Mg limitation impairs IAA synthesis. Consistently, IAA accumulation decreased by ∼23% in the root in contrast to the ∼40% increase in the shoot under Mg limitation (Figure 6A), clearly suggesting that Mg limitation reshaped auxin synthesis and distribution pattern between above- and below-ground. Such significant lower levels of auxin ultimately converted into developmental cues to slow down root growth.

**FIGURE 8:**
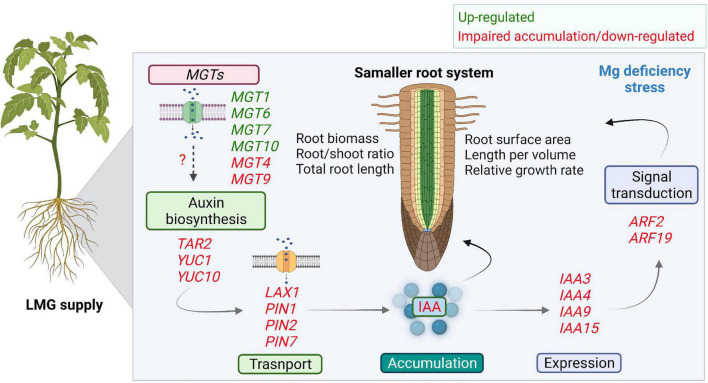
Scheme depicting the impaired auxin accumulation and signaling in Mg-deficient root. Differential expression of *MGTs* in the root favors Mg uptake and translocation under Mg limitation. Low Mg leads to smaller root related indexes. In the root, Mg limitation down-regulates gene expression along auxin synthesis, transport, and signal transduction. Where, *MGTs*, Mg transporters; YUC, YUCCA; LAX, LIKE-AUX1; PIN, PIN-FORMED; Aux/IAA, AUXIN/INDOLE ACETIC ACID; ARFs, AUXIN RESPONSE FACTORS.

Auxin transport and signaling components play essential roles in root system development (Porco et al., 2016; Du and Scheres, 2018; Hu et al., 2021). AUX/LAX, PIN2, PIN3, and PIN7 all participate in root development (Paponov et al., 2005; Marhavy et al., 2013). Our results showed significant transcriptional repression of *LAX1*, *PIN1*, *PIN2*, and *PIN7* in the LMG root (Figure 7A), in agreement with lower levels of auxin. Aux/IAA is a key regulator of auxin-modulated signal transduction (Chandler, 2016; Israeli et al., 2020; Lv et al., 2020). In our study, expression of *IAA3*, *IAA4*, *IAA9*, and *IAA15* was depressed in the LMG root (Figure 7D). Further, *ARF2* and *ARF19* expression was highly down-regulated in the root under Mg limitation (Figure 7F). The transcriptional activators ARF7 and ARF19 play an important role in root branching (Lee et al., 2019). Based on the actual expression levels, it is speculated that the relative strong expressed genes such as *MGT7*, *TAR2*, *YUC1*, *PIN1*, *PIN2*, *PIN7*, *IAA4*, *IAA15*, and *ARF2* may altered the auxin-related process in Mg-deficient roots. Hence, on the basis of consistent decreases in auxin accumulation and attenuation of related gene expression along auxin signaling in the LMG root (Figures 6, 7), we propose that LMG led to weakened auxin functioning, i.e., synthesis, transport, accumulation, and signal transduction in the root system (Figure 8).

The work presented in this study provides a new valuable insight that LMG supply disturbs transcription of auxin signaling genes, which well fit in the phenotype of smaller roots. However, in future detailed molecular investigation with mutant lines and protein functions is required to directly link such phenotypes to auxin signaling. In summary, given the crucial roles of auxin in modulating root growth, it is speculated that down-tuning of auxin accumulation and signaling in the root preconditions smaller root related indexes, while expression of MGTs are mostly up-regulated for Mg uptake and translocation.

## Conclusion

Phylogenetic analysis suggested that Mg transporters (MRS2/MGTs) constitute a previously uncharacterized 3-clade tree *in planta*. In adaptation to internal Mg deficiency, tomato seedlings altered *MGTs* expression under Mg limitation. Notably, lower auxin (IAA) accumulation in Mg-deficient roots was consistent with systemic down-tuning of gene expression in auxin synthesis (TAR/*YUCs*), transport (*LAXs, PINs*), and signaling (*IAAs, ARFs*). Given the crucial roles of auxin in modulating root growth, it is speculated that weakened auxin functioning under Mg limitation may precondition a smaller root system. Further efforts are required to better understand the molecular functioning of MRS2/MGTs, auxin signaling and its involvement in modulating root growth under Mg limitation.

## Data Availability Statement

The datasets presented in this study can be found in online repositories. The names of the repository/repositories and accession number(s) can be found in the article/Supplementary Material.

## Author Contributions

MI and XL conceived, designed the study, wrote, and revised the manuscript. MI and YW performed the experiments. MI and YZ analyzed the data. All authors have reviewed and approved the submitted version.

## Conflict of Interest

The authors declare that the research was conducted in the absence of any commercial or financial relationships that could be construed as a potential conflict of interest.

## Publisher’s Note

All claims expressed in this article are solely those of the authors and do not necessarily represent those of their affiliated organizations, or those of the publisher, the editors and the reviewers. Any product that may be evaluated in this article, or claim that may be made by its manufacturer, is not guaranteed or endorsed by the publisher.

## Footnotes

1
https://phytozome.jgi.doe.gov

2
https://blast.ncbi.nlm.nih.gov/Blast.cgi

3
http://www.drive5.com/muscle/

4
https://www.softpedia.com/get/Science-CAD/GeneDoc.shtml

5
http://www.clustal.org/clustal2/

6
https://www.megasoftware.net/history.php

7
http://gsds.cbi.pku.edu.cn/
