# Supplementary material for: Magnesium Limitation Leads to Transcriptional Down-Tuning of Auxin Synthesis, Transport, and Signaling in the Tomato Root
Source: Front Plant Sci. 2021 Dec 23;12:802399. doi: 10.3389/fpls.2021.802399 (PMC8733655; doi:10.3389/fpls.2021.802399)
Supplement: Supplementary file 1 [file Table_1.DOCX]

Supplementary Material

**Table S1.** The accession numbers of genes used for phylogenetic analysis and gene structure.

| Name | Accession No. | Species | Family | Order | Database |
| --- | --- | --- | --- | --- | --- |
| AmMGT1 | AmTr_v1.0_scaffold00019.13 | Amborella trichopoda | Amborellaceae | Amborellales | Phytozome |
| AmMGT10 | AmTr_v1.0_scaffold00002.387 | Amborella trichopoda | Amborellaceae | Amborellales | Phytozome |
| AmMGT2a | AmTr_v1.0_scaffold00019.17 | Amborella trichopoda | Amborellaceae | Amborellales | Phytozome |
| AmMGT2b | AmTr_v1.0_scaffold00019.18 | Amborella trichopoda | Amborellaceae | Amborellales | Phytozome |
| AmMGT3 | AmTr_v1.0_scaffold00154.12 | Amborella trichopoda | Amborellaceae | Amborellales | Phytozome |
| AmMGT4a | AmTr_v1.0_scaffold00065.95 | Amborella trichopoda | Amborellaceae | Amborellales | Phytozome |
| AmMGT4b | AmTr_v1.0_scaffold00017.109 | Amborella trichopoda | Amborellaceae | Amborellales | Phytozome |
| AmMGT4c | AmTr_v1.0_scaffold00017.108 | Amborella trichopoda | Amborellaceae | Amborellales | Phytozome |
| AmMGT4d | AmTr_v1.0_scaffold00017.107 | Amborella trichopoda | Amborellaceae | Amborellales | Phytozome |
| AmMGT6a | AmTr_v1.0_scaffold00008.22 | Amborella trichopoda | Amborellaceae | Amborellales | Phytozome |
| AmMGT6b | AmTr_v1.0_scaffold00056.31 | Amborella trichopoda | Amborellaceae | Amborellales | Phytozome |
| AmMGT9 | AmTr_v1.0_scaffold00002.503 | Amborella trichopoda | Amborellaceae | Amborellales | Phytozome |
| AtMGT1 | AT1G80900.1 | Arabidopsis thaliana | Brassicaceae | Brassicales | Phytozome |
| AtMGT10 | AT5G22830.1 | Arabidopsis thaliana | Brassicaceae | Brassicales | Phytozome |
| AtMGT2 | AT1G16010.2 | Arabidopsis thaliana | Brassicaceae | Brassicales | Phytozome |
| AtMGT3 | AT2G03620.1 | Arabidopsis thaliana | Brassicaceae | Brassicales | Phytozome |
| AtMGT4 | AT3G19640.1 | Arabidopsis thaliana | Brassicaceae | Brassicales | Phytozome |
| AtMGT5 | AT4G28580.1 | Arabidopsis thaliana | Brassicaceae | Brassicales | Phytozome |
| AtMGT6 | AT3G58970.1 | Arabidopsis thaliana | Brassicaceae | Brassicales | Phytozome |
| AtMGT7 | AT5G09690.2 | Arabidopsis thaliana | Brassicaceae | Brassicales | Phytozome |
| AtMGT8a | AT5G09720.1 | Arabidopsis thaliana | Brassicaceae | Brassicales | Phytozome |
| AtMGT8b | AT5G09710.1 | Arabidopsis thaliana | Brassicaceae | Brassicales | Phytozome |
| AtMGT9 | AT5G64560.1 | Arabidopsis thaliana | Brassicaceae | Brassicales | Phytozome |
| CrMGT10 | Cre02.g144006.t1.1 | Chlamydomonas reinhardtii | Chlamydomonadaceae | Chlamydomonadales | Phytozome |
| CrMGT1a | Cre12.g550277.t1.1 | Chlamydomonas reinhardtii | Chlamydomonadaceae | Chlamydomonadales | Phytozome |
| CrMGT1b | Cre07.g352750.t1.2 | Chlamydomonas reinhardtii | Chlamydomonadaceae | Chlamydomonadales | Phytozome |
| CrSmMRS2 | Cre50.g761497.t1.1 | Chlamydomonas reinhardtii | Chlamydomonadaceae | Chlamydomonadales | Phytozome |
| MpMGT1 | Mapoly0071s0010.1 | Marchantia polymorpha | Marchantiaceae | Marchantiales | Phytozome |
| MpMGT10 | Mapoly0034s0035.1 | Marchantia polymorpha | Marchantiaceae | Marchantiales | Phytozome |
| MpMGT4 | Mapoly0118s0011.1 | Marchantia polymorpha | Marchantiaceae | Marchantiales | Phytozome |
| MpMGT6 | Mapoly0097s0060.1 | Marchantia polymorpha | Marchantiaceae | Marchantiales | Phytozome |
| MpMGT9 | Mapoly0001s0270.1 | Marchantia polymorpha | Marchantiaceae | Marchantiales | Phytozome |
| MpMRS2 | Mapoly0032s0166.1 | Marchantia polymorpha | Marchantiaceae | Marchantiales | Phytozome |
| OsMGT1 | LOC_Os04g42280.1 | Oryza sativa | Poaceae | Poales | Phytozome |
| OsMGT10 | LOC_Os03g48000.1 | Oryza sativa | Poaceae | Poales | Phytozome |
| OsMGT2 | LOC_Os06g44150.1 | Oryza sativa | Poaceae | Poales | Phytozome |
| OsMGT4a | LOC_Os01g68040.1 | Oryza sativa | Poaceae | Poales | Phytozome |
| OsMGT4b | LOC_Os01g64890.1 | Oryza sativa | Poaceae | Poales | Phytozome |
| OsMGT4c | LOC_Os04g35160.1 | Oryza sativa | Poaceae | Poales | Phytozome |
| OsMGT6a | LOC_Os03g04480.1 | Oryza sativa | Poaceae | Poales | Phytozome |
| OsMGT6b | LOC_Os10g39790.2 | Oryza sativa | Poaceae | Poales | Phytozome |
| OsMGT9 | LOC_Os03g53110.1 | Oryza sativa | Poaceae | Poales | Phytozome |
| PpMGT10a | Pp3c5_14300V3.1 | Physcomitrella patens | Funariaceae | Funariales | Phytozome |
| PpMGT10b | Pp3c25_90V3.1 | Physcomitrella patens | Funariaceae | Funariales | Phytozome |
| PpMGT1a | Pp3c8_23170V3.1 | Physcomitrella patens | Funariaceae | Funariales | Phytozome |
| PpMGT1b | Pp3c24_14620V3.1 | Physcomitrella patens | Funariaceae | Funariales | Phytozome |
| PpMGT4a | Pp3c2_23840V3.1 | Physcomitrella patens | Funariaceae | Funariales | Phytozome |
| PpMGT6a | Pp3c4_21750V3.1 | Physcomitrella patens | Funariaceae | Funariales | Phytozome |
| PpMGT6b | Pp3c3_5770V3.1 | Physcomitrella patens | Funariaceae | Funariales | Phytozome |
| PpMGT6c | Pp3c12_6460V3.1 | Physcomitrella patens | Funariaceae | Funariales | Phytozome |
| PpMGT9 | Pp3c2_34940V3.1 | Physcomitrella patens | Funariaceae | Funariales | Phytozome |
| PpMRS2a | Pp3c11_4790V3.1 | Physcomitrella patens | Funariaceae | Funariales | Phytozome |
| PpMRS2a | Pp3c3_33028V3.1 | Physcomitrella patens | Funariaceae | Funariales | Phytozome |
| ScMFM1 | GAX68793.1 | Saccharomyces cerevisiae | Schizosaccharomycetaceae | Schizosaccharomycetales | NCBI |
| ScMfm1p | PJP09556.1 | Saccharomyces cerevisiae | Schizosaccharomycetaceae | Schizosaccharomycetales | NCBI |
| ScMRS2a | AAA34795.1 | Saccharomyces cerevisiae | Schizosaccharomycetaceae | Schizosaccharomycetales | NCBI |
| ScMRS2b | AAT93188.1 | Saccharomyces cerevisiae | Schizosaccharomycetaceae | Schizosaccharomycetales | NCBI |
| SlMGT1 | Solyc06g068490.2.1 | Solanum lycopersicum | Solanaceae | Solanales | Phytozome |
| SlMGT10 | Solyc11g066660.1.1 | Solanum lycopersicum | Solanaceae | Solanales | Phytozome |
| SlMGT3 | Solyc05g012220.2.1 | Solanum lycopersicum | Solanaceae | Solanales | Phytozome |
| SlMGT4 | Solyc01g106900.2.1 | Solanum lycopersicum | Solanaceae | Solanales | Phytozome |
| SlMGT6 | Solyc01g103890.2.1 | Solanum lycopersicum | Solanaceae | Solanales | Phytozome |
| SlMGT7 | Solyc03g005390.2.1 | Solanum lycopersicum | Solanaceae | Solanales | Phytozome |
| SlMGT9 | Solyc09g065920.2.1 | Solanum lycopersicum | Solanaceae | Solanales | Phytozome |
| SmMGT1 | Sm165647 | Selaginella moellendorffii | Selaginellaceae | Selaginellales | Phytozome |
| SmMGT10 | Sm231708 | Selaginella moellendorffii | Selaginellaceae | Selaginellales | Phytozome |
| SmMGT2 | Sm165091 | Selaginella moellendorffii | Selaginellaceae | Selaginellales | Phytozome |
| SmMGT4 | Sm53758 | Selaginella moellendorffii | Selaginellaceae | Selaginellales | Phytozome |
| SmMGT6 | Sm76370 | Selaginella moellendorffii | Selaginellaceae | Selaginellales | Phytozome |
| SmMGT9a | Sm422512 | Selaginella moellendorffii | Selaginellaceae | Selaginellales | Phytozome |
| SmMGT9b | Sm426446 | Selaginella moellendorffii | Selaginellaceae | Selaginellales | Phytozome |
| SmMGT9c | Sm91491 | Selaginella moellendorffii | Selaginellaceae | Selaginellales | Phytozome |
| SmMRS2a | Sm431872 | Selaginella moellendorffii | Selaginellaceae | Selaginellales | Phytozome |
| SmMRS2b | Sm429777 | Selaginella moellendorffii | Selaginellaceae | Selaginellales | Phytozome |
| SpMRS2 | SpMRS2 | Schizosaccharomyces pombe | Schizosaccharomycetaceae | Schizosaccharomycetales | NCBI |
| ZmMGT1 | GRMZM2G064467 | Zea mays | Poaceae | Poales | Phytozome |
| ZmMGT10 | GRMZM2G420436 | Zea mays | Poaceae | Poales | Phytozome |
| ZmMGT2a | GRMZM2G108477 | Zea mays | Poaceae | Poales | Phytozome |
| ZmMGT2b | GRMZM2G170326 | Zea mays | Poaceae | Poales | Phytozome |
| ZmMGT4a | GRMZM2G139822 | Zea mays | Poaceae | Poales | Phytozome |
| ZmMGT4b | GRMZM2G065971 | Zea mays | Poaceae | Poales | Phytozome |
| ZmMGT4c | GRMZM2G458879 | Zea mays | Poaceae | Poales | Phytozome |
| ZmMGT4d | GRMZM2G054632 | Zea mays | Poaceae | Poales | Phytozome |
| ZmMGT6a | GRMZM2G018706 | Zea mays | Poaceae | Poales | Phytozome |
| ZmMGT6b | GRMZM2G145794 | Zea mays | Poaceae | Poales | Phytozome |
| ZmMGT6c | GRMZM2G453832 | Zea mays | Poaceae | Poales | Phytozome |
| ZmMGT9 | GRMZM2G159295 | Zea mays | Poaceae | Poales | Phytozome |

**Table S2.** The primers information used for RT-qPCR analysis.

| Gene symbols | Descriptions | Sequence (5’ to 3’) |
| --- | --- | --- |
| *SlMGT1* | Magnesium transporter 1 | F: GACAAGTTCACTATGATGCGTC  R: ACTACATACTGCAGGACATAGC |
| *SlMGT3* | Magnesium transporter 3 | F: GGTCATGGGAGTCGCTCTTG  R: CTTAGGGTCCAAAAGCCGGA |
| *SlMGT4* | Magnesium transporter 4 | F: CGATTTAGCTTATCCCGGAGAT  R: GAATCCAACAGAAGCCATGAAC |
| *SlMGT6* | Magnesium transporter 6 | F: GCGAATCAGTGGAAGAAGAATC  R: CTTCCTACGGCGAATAGAGAAT |
| *SlMGT7* | Magnesium transporter 7 | F: GTGGCACCTTACTGGTCTCC  R: ACAAGACCCTTGTGCCTAGC |
| *SlMGT9* | Magnesium transporter 9 | F: CACGCTCGTGATCTTCGGAT  R: TCATTGGCTTCAGTCGCCTT |
| *SlMGT10* | Magnesium transporter 10 | F: TGAGAGCTCGGAAGGTGAGA  R: AGAAGCACTCCTCTGCGAAC |
| SlTAR2 | Tryptophan Aminotransferase-Related | F: TTTGCAACACAGCCAGCTTTTG  R: CCCCGCCTCTTGTCAATATC |
| *SlYU1* | YUCCA 1 | F: TGGACATTGGTGCATTGGAA  R: AGCCAGTAGCAAGAAGAACAGA |
| *SlYUC2/6* | YUCCA 2/6 | F: CCTCGTGGCTAAAGGAAAAAG  R: CACTGCATAAAGTCCACACTC |
| *SlYUC8* | YUCCA 8 | F: GGGAGATGGTGTACATGGCAA  R: AATGGGCCTTCTTCTGGTTGT |
| *SlYUC10* | YUCCA 10 | F: GAGCTGGACCATCCGGTATC  R: AAAGGCCGCTTCAACACAAG |
| *SlIAA3* | Indole-3-acetic acid 3 | F: GCCACCAGTTCGATCATACA  R: ATAAGGTGCTCCATCCATGC |
| *SlIAA4* | Indole-3-acetic acid 4 | F: ACTCCACCTGTTGCCAAGAC  R: AGATAAGGGGCTCCATCCAT |
| *SlIAA9* | Indole-3-acetic acid 9 | F: CAGAGGGGAAGTTTCTGTCG  R: CAACCTGTGCCTTTGTAGCA |
| *SlIAA15* | Indole-3-acetic acid 15 | F: ATCGGAGACAGCCAAATCAG  R: TTTGCTGGAGGTTTGTTTCC |
| *SlLAX1* | LIKE-AUX1 | F: GTTGGACTGCTATGTATGTTATC  R: TGCTGGTGGAAGTGAAGG |
| *SlPIN1* | PIN-FORMED 1 | F: GCTGCAGGCTGGTCTAGATT  R: AACAATGGCAACAAAGCACA |
| *SlPIN2* | PIN-FORMED 2 | F: CGAGGAGGTAGGAGTATGAG  R: CACTTCCGCTTCCAACTTC |
| *SlPIN3* | PIN-FORMED 3 | F: GCTGCCGCTTCTATTATCG  R: CTCCTTAGCAAACACAAATGG |
| *SlPIN4* | PIN-FORMED 4 | F: AGTTATGGCTGCTGCTTC  R: CAAACACAAATGGGACAATC |
| *SlPIN7* | PIN-FORMED 3 | F: ATCAGCGGTCCAGCAGTC  R: GAACGATTCCTTGAGGTAGAGC |
| *SlARF2* | Auxin response factor 2 | F: GCAAGGTCAAGAGTTATCGA  R: CATTGGTTTCTCAGACAAGTC |
| *SlARF6* | Auxin response factor 6 | F: AGTGTCTCCTTCCTCATCATC  R: ATTGTTTTGGCTAACTGCTAC |
| *SlARF8* | Auxin response factor 8 | F: TCGAATGCGGACGTTTACC  R: TAGCCTGAGTAACGTGCGATGT |
| *SlARF19* | Auxin response factor 19 | F: AATGGCTTCCGATTATGTCACC  R: TCCTACTGCACCACGCTTGTAC |

**Figure S1. Mg depletion inhibited root architectural indexes after two weeks of onset of treatments.** (A) scanned image of root under control and LMG application, (B) root biomass (mg plant^-1^ DM), (C) root-to-shoot ratio (DM), (D) total root length (cm), (E) total root surface area (cm^2^), (F) root length per volume (cm m^-3^), and (G) average root diameter (mm). The bar graph shows mean value while whiskers represent the maximum/minimum values of six independent biological replicates. Asterisks indicate a significant difference at *P < 0.05 according to Tukey’s HSD test. Where, Ctrl, control; LMG, low Mg; ns, non-significant
